# *Ab initio* structure determination of n-diamond

**DOI:** 10.1038/srep13447

**Published:** 2015-08-24

**Authors:** Da Li, Fubo Tian, Binhua Chu, Defang Duan, Xiaojing Sha, Yunzhou Lv, Huadi Zhang, Nan Lu, Bingbing Liu, Tian Cui

**Affiliations:** 1 State Key Lab of Superhard Materials, College of Physics, Jilin University, Changchun 130012, P. R. China; 2 State Key Lab of Supramolecular Structure and Materials, College of Chemistry, Jilin University, Changchun 130012, P. R. China

## Abstract

A systematic computational study on the crystal structure of n-diamond has been performed using first-principle methods. A novel carbon allotrope with hexagonal symmetry *R*32 space group has been predicted. We name it as *HR*-carbon. *HR*-carbon composed of lonsdaleite layers and unique *C*_3_ isosceles triangle rings, is stable over graphite phase above 14.2 GPa. The simulated x-ray diffraction pattern, Raman, and energy-loss near-edge spectrum can match the experimental results very well, indicating that *HR*-carbon is a likely candidate structure for n-diamond. *HR*-carbon has an incompressible atomic arrangement because of unique *C*_3_ isosceles triangle rings. The hardness and bulk modulus of *HR*-carbon are calculated to be 80 GPa and 427 GPa, respectively, which are comparable to those of diamond. *C*_3_ isosceles triangle rings are very important for the stability and hardness of *HR*-carbon.

The graphite composed of *sp*^2^-hybridized bonds is one of the most stable carbon phases in nature. Under high pressure, the rehybridization from *sp*^2^-bonding to *sp*^3^-bonding appears in graphite. It can transform to *sp*^3^-bonding diamond under high pressure (15 GPa) and high temperatures (1600–2500 K) without using the catalysts^1^,^2^. Under room temperature, the graphite converts to distorted *sp*^3^-bonding cold-compressed post graphite phase at above 14 GPa^3^. The transformation mechanisms of diamond/cold-compressed graphite phase transition have been studied systematacially^4^,^5^. The possible crystal structures of cold-compressed post graphite phases have been explored by both theoretical and experimental methods, such as M-carbon, W*-*carbon, C-carbon etc^6^,^7^,^8^,^9^,^10^,^11^,^12^,^13^,^14^,^15^,^16^,^17^,^18^,^19^,^20^,^21^,^22^,^23^,^24^,^25^. By using shock compression and rapid quenching methods, the graphite transforms to a new diamond-like phase (n-diamond)^26^ which has also been confirmed by several other experimental techniques^27^,^28^,^29^,^30^,^31^,^32^,^33^,^34^,^35^. Non-hydrostatic pressure shows a much more significant effect on the formation of n-diamond. The n-diamond is a metastable phase of graphite as diamond and can also be synthesized from other carbon forms such as carbon black, nanotubes, C_60_ films etc^35^,^36^,^37^. So far, although n-diamond has been found for more than thirty years, the crystal structure of n-diamond is still mysterious. X-ray powder diffraction (XRD) has already been used to characterize n-diamond. Previous observations indicate that the XRD of n-diamond is very similar to that of cubic diamond^27^. Interestingly, some forbidden reflections of cubic diamond have also been observed at the same time in experiments. The n-diamond was initially thought to be a face-central cubic modified diamond (Fcc carbon)^31^,^32^,^38^,^39^,^40^. However, theoretical calculations indicate that the Fcc carbon is less stable than the other candidate phases^41^,^42^,^43^. Moreover, the energy-loss near-edge spectrum (ELNES) of Fcc carbon do not agree with the measured ELNES spectrum^44^,^45^. The carbon-hydrogen zincblende structure, hydrogen-doped cubic diamond, intermediate phases between graphite and diamond with cubic *F*43*m* or rhombohedral *R*3 space group and tetragonal glitter carbon have also been proposed as the candidates for n-diamond^46^,^47^,^48^,^49^. However, only glitter carbon is dynamically stable phase among the known candidate structures^47^. Moreover, to our knowledge, none of them can account for the experimental XRD very well. In addition, experimental samples are usually a mixture of different reactants, making the comparison between experimental and theoretical XRD even harder. Up to now, the crystal structure of n-diamond is the subject of continuing debate. Hear we predicted a likely candidate structure for n-diamond by using the genetic algorithm for crystal structure prediction. It has *R*32 space group and is more energetically favorable than the other candidates. The simulated XRD patterns, Raman and ELNES spectra can match the experimental data. We call this novel hexagonal carbon allotrope as *HR*-carbon which has an incompressible atomic arrangement due to unique *C*_3_ isosceles triangle rings. It is stable in the range of 0 GPa up to 50 GPa at least. *C*_3_ isosceles triangle rings of *HR*-carbon are critical for the stability and hardness of *HR*-carbon.

## Results and Discussion

**Figure 1:**
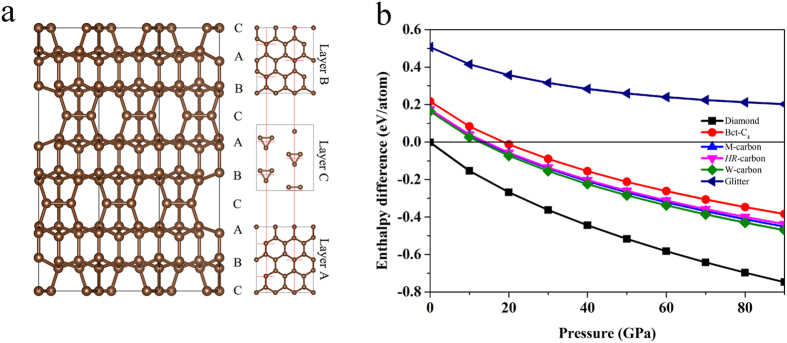
The structure of *HR*-carbon and enthalpy difference curves. (**a**) The crystal structure of *HR*-carbon and side views of *A*, *B* and *C* Layer from the [001] direction. (**b**) The enthalpy per atom of *HR*-carbon and other carbon polymorphs as functions of pressure with respect to graphite.

**Figure 2:**
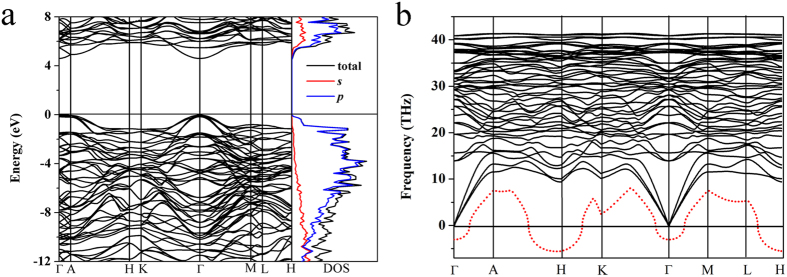
The electronic band structure, partial density of state and phonon spectra of *HR*-carbon. (**a**) Calculated electronic band structure and partial density of state of *HR*-carbon. (**b**) The phonon spectra of *HR*-carbon at ambient pressure. The red dash line is the imaginary phonon mode of modulated *HR-carbon*.

A novel candidate structure for n-diamond, named it as *HR*-carbon, has been predicted by using USPEX method^50^,^51^,^52^ with 15 carbon atoms in the simulation cell. *HR*-carbon has hexagonal lattice *R*32 symmetry as depicted in Fig. 1(a). It is made of exclusively three-dimensional *sp*^3^-hybridized covalent bonds. The equilibrium lattice parameters are *a* = 4.305 Å, *c* = 16.291 Å at ambient condition. Within this structure, five inequivalent atoms occupy the crystallographic 18*f* and 6*c* sites in the unit cell, which are (0.667, 0.009, 0.247), (0.667, 0.333, 0.114), (0.0, 0.0, 0.127), (0.333, 0.667, 0.117) and (0.871, 0.538, 0.333) positions. There are 45 atoms in the unit cell. In rhombohedral representation, it has a 15 atoms rhombohedral unit cell. The crystal structure of *HR*-carbon is distinct from that of diamond. The *HR*-carbon can be regarded as a modulated graphite phase composed of lonsdaleite layers and *C*_3_ isosceles triangle rings layers with stacking sequence of *ABCABCABC*…along the crystallographic *c* axis of hexagonal lattice. The close-packed *A* and *B* layers are lonsdaleite layers. The *C* layers composed of isolated *C*_3_ isosceles triangle rings are sandwiched between two *AB* layers with unique twisted *sp*^3^ covalent bonds with bond angle 60°, 107.15°, and 140.04° which are much different from those of standard *sp*^3^ covalent bonds of diamond (bond angle 109.47°). This finding suggests a novel combination form of carbon atoms to construct post graphite phases. The structural type of *HR*-carbon is also consistent with previous theoretical suggestion that n-diamond should have cubic or rhombohedral space group^46^. Enthalpy calculations suggest that *HR*-carbon is much stable than the previously proposed candidates of n-diamond Fig. 1(b)^47^. And *HR*-carbon becomes stable relative to graphite at above 14.2 GPa which is almost equal to the phase transition pressure of cold-compressed graphite phase (14 GPa)^3^. Its bulk modulus and shear modulus are 428 and 471 GPa, respectively. Furthermore, no imaginary frequencies are observed throughout the whole Brillouin zone in phonon dispersion Fig. 2(b), confirming dynamically structural stability of *HR*-carbon. The highest phonon frequency of *HR*-carbon (41.5 THz) is very close to that of diamond (40 THz)^53^, which reflects the diamond-like structural bonding character of *HR*-carbon. The calculated electronic band structure and density of state (DOS) at ambient pressure reveal that the *HR*-carbon is semiconductor with direct band gap 4.6 eV Fig. 2(a). Strong hybridization between *s* and *p* orbitals of DOS indicates the presence of strong covalent bonds in *HR*-carbon. The *HR*-carbon can be expected to have good mechanical properties among compressed graphite phases.

**Figure 3:**
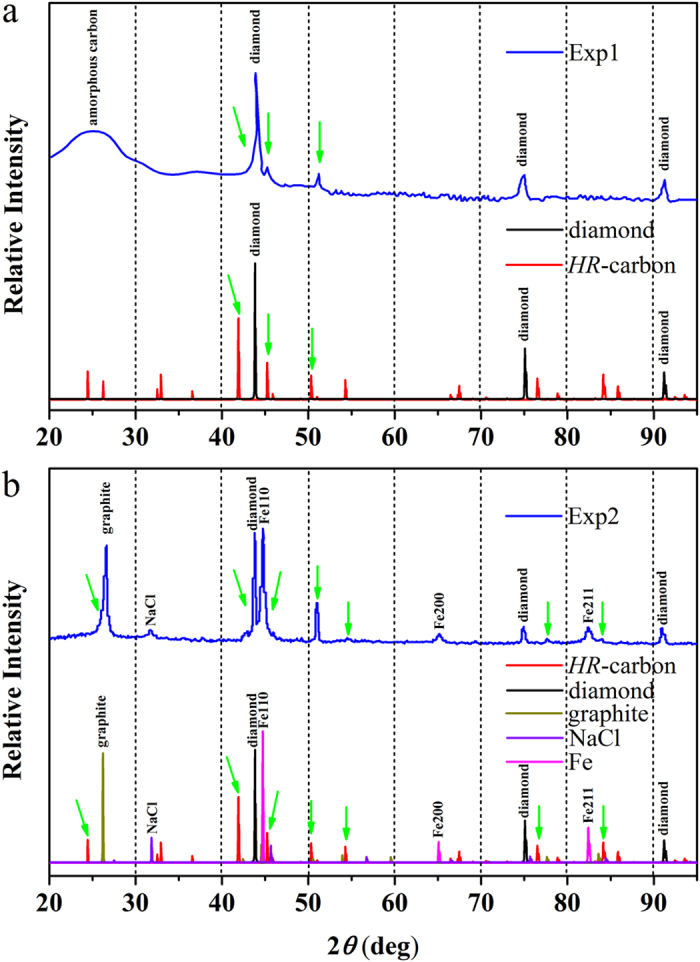
The comparison of theoretical and experimental XRD. (**a**) The simulated XRD patterns of *HR*-carbon, diamond, and experimental XRD pattern (from Ref. 27) (**b**) The simulated XRD patterns of *HR*-carbon, diamond, graphite, NaCl, α-Fe and experimental XRD pattern (from Ref. 49). The used x-ray wavelength is 1.5405 Å as employed in the experiments. Green arrows stand for those XRD peaks of *HR*-carbon observed by two experiments.

**Figure 4:**
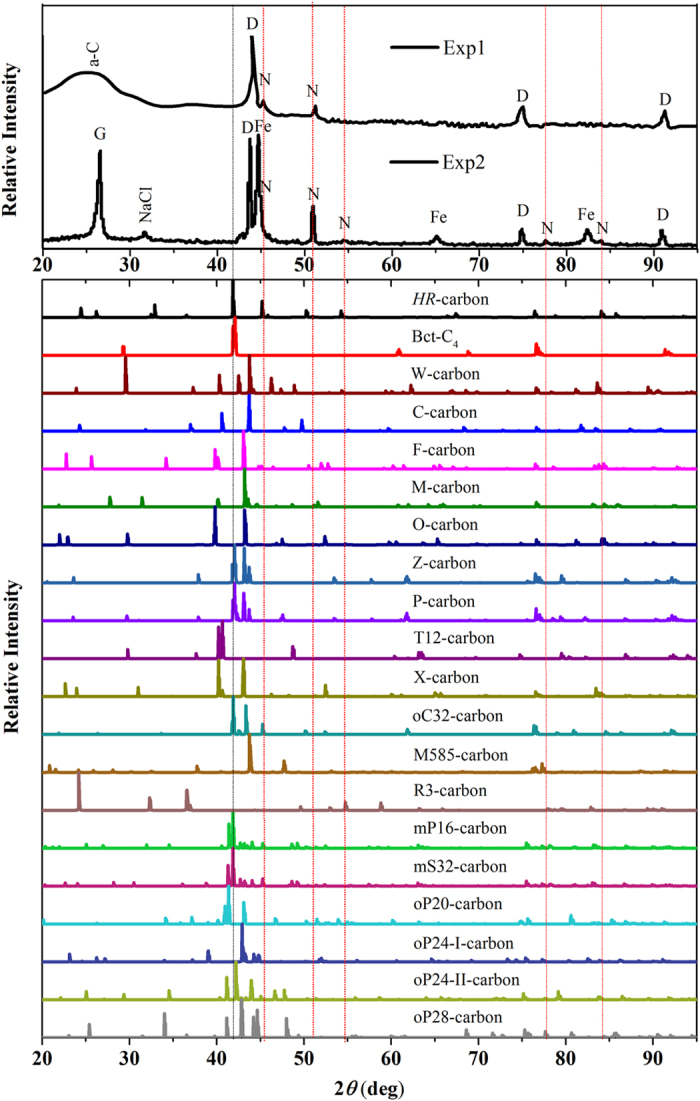
The XRD patterns of experiments, *HR*-carbon and previously proposed post-graphite phases. The XRD of experiment 1 comes from Ref. 27. The XRD of experiment 2 comes from Ref. 49. a-C: amorphous carbon; G: graphite; N: n-diamond; D: diamond; Fe: iron.

To confirm the consistency of *HR*-carbon and n-diamond, we simulate the XRD pattern of *HR*-carbon and compare with the experimental results Fig. 3^27^,^49^. Our simulated XRD patterns for *HR*-carbon, reactants, and products are consistent with experimental XRD patterns. Three individual peaks of diamond can be easily indexed in the experimental XRD pattern Fig. 3(a). And the peaks of *HR*-carbon at ~46° and 51° can explain the experimentally observed forbidden diamond peaks. The relatively weaker peaks of *HR*-carbon in the range of 54°–95° have merged into the background. While the peaks at ~42°, 76° and 93° contribute to the peaks broadening of diamond at ~45°, 75° and 90°. The peaks of *HR*-carbon in the range of 20°–40° have been covered by the peaks of amorphous carbon (a-C). Furthermore, we also compare our simulated XRD pattern with that of n-diamond synthesized by Fe-catalysed carbon black methods^50^. The peaks of diamond, graphite, NaCl, and Fe have been indexed in the experimental XRD pattern Fig. 3(b). Four characteristic peaks of experimental XRD at 50°, 54°, 77°, and 84° can be explained very well by the peaks of *HR*-carbon in the same range. The peaks of *HR*-carbon at ~24° and 26°, ~33°, and ~45° contribute the peak broadening of graphite (~26°), NaCl (~32°) and Fe (~45°), respectively. The peaks of *HR*-carbon at ~42° can explain the experimentally observed shoulder peak at ~43°. The other peaks of *HR*-carbon have merged to the background. It is noted that the other candidate structures can also simulate the experimental XRD patterns. However, they are all thermodynamically unstable with respect to *HR*-carbon. We also compare the XRD pattern of *HR*-carbon with those of previously proposed post-graphite phases as shown in Fig. 4. Nineteen inequivalent structures (M-carbon^7^, W-carbon^11^, Bct-C_4_^8^, X-carbon^18^, O-carbon^21^ (H-carbon^20^, R-carbon^19^), C-carbon^16^ (S-carbon^20^), F-carbon^12^ (J-carbon^22^), T12-carbon^13^, Z-carbon^15^ (Cco-C8^9^, oC16-II^10^), P-carbon^19^ (Z4-A3B1^14^), oC32-carbon^23^, M585-carbon^25^, R3-carbon^24^, mP16-carbon^6^, mS32-carbon^6^, oP20-carbon^6^, oP24-I-carbon^6^, oP24-II-carbon^6^ and oP28-carbon^6^) have been considered. It can be found that only the XRD of *HR*-carbon at ~45°, ~50°, ~54°, ~76° and ~84° can explain the experimentally observed characteristic peaks of n-diamond (45°, 51°, 55°, 78° and 84°)^27^,^49^. So *HR*-carbon is the most possible candidate structure of n-diamond.

**Figure 5:**
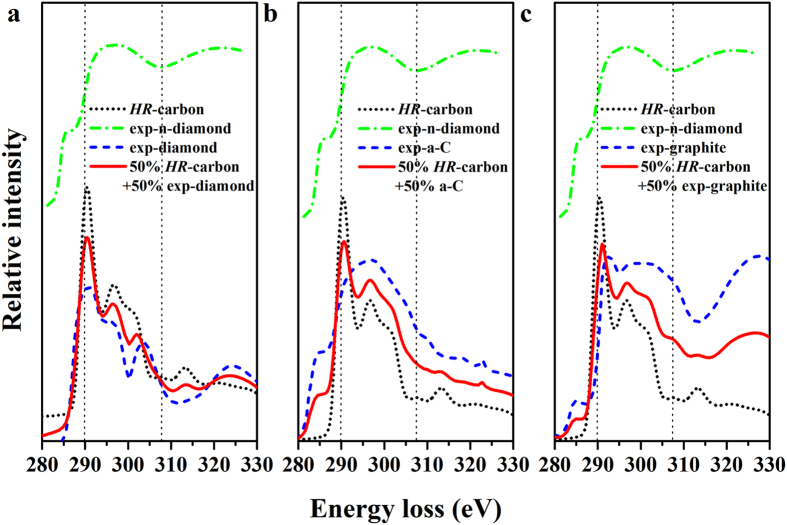
The comparison of theoretical and experimental ELNES. Theoretical ELNES of *HR*-carbon, experimental ELNES of diamond, graphite, n-diamond and a-C and (**a**) the fitting ELNES of the mixture of 50% *HR*-carbon + 50% diamond (**b**) the fitting ELNES of the mixture of 50% *HR*-carbon + 50% amorphous carbon. (**c**) the fitting ELNES of the mixture of 50% *HR*-carbon + 50% graphite. Experimental data come from Ref. 32.

**Figure 6:**
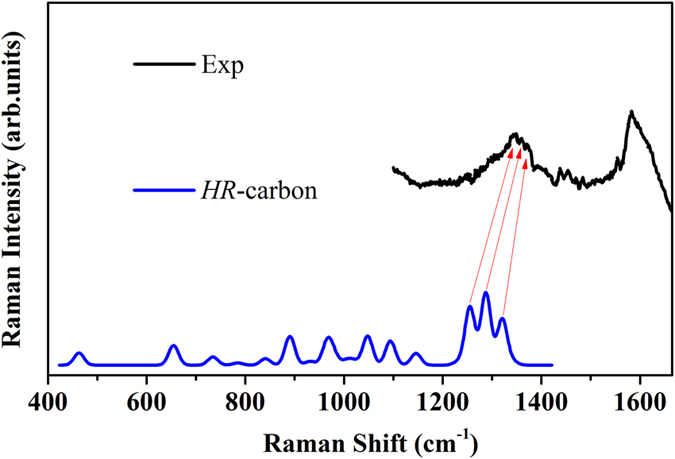
The comparison of our simulated Raman of *HR*-carbon and experimental data. The experimental data come from Ref. 38.

To confirm reliability of our structure, the ELNES and Raman of *HR*-carbon was calculated and compared with the experimental results^32^,^38^. The ELNES spectra are very useful to distinguish the various hybridized covalent bonds in carbon materials. Strikingly, the XRD results indicate a coexistence of n-diamond, amorphous carbon, diamond and some other carbon allotropes, consistent with Konyashin’s conclusion that the experimental sample is a mixture of n-diamond and other carbon allotropes^44^. Obvious π* feature of experimental ELNES indicates the presence of *sp*^2^-hybridized covalent bonds. However, no π* features are found in the ELNES of diamond and *HR*-carbon, indicating the sample should contain graphite or a-C because those are composed of *sp*^2^-hybridized covalent bonds. Moreover, the a-C was already detected by XRD because of the presence of broad peak in range of 20–30^°^ Fig. 3(a). In order to compare to experimental data, we calculate the fitting average values (FAV) of *HR*-carbon and possible carbon allotropes (diamond, graphite and amorphous carbon) (Fig. 5). It can be found that the FAV of *HR*-carbon and diamond cannot match the experimental ELNES. The FAV of *HR*-carbon and graphite/a-C are very similar to each other. The two main differences between those two spectra are found in range of 280–290 eV and 315–330 eV. Obvious π* feature can be found in the range of 280–290 eV in those two situation. However, the intensity of π* peak of FAV of *HR*-carbon and a-C is stronger than that of π* peak of FAV of *HR*-carbon and graphite and can match the experimental data very well. But only the peaks of FAV of *HR*-carbon and graphite in the range of 315–330 eV can match the experimental third broad peak. It is noteworthy that the presence of graphite was not reported in Li’s experimental XRD pattern^27^ because the main peaks of graphite and amorphous carbon merge together in the XRD pattern and some weak peaks have been covered by the background. Moreover, only the valley of ELNES of *HR*-carbon in range of 305–310 eV can explain the experimental valley between the second and third board peaks at 310 eV very well. This valley is an important key to confirm the existence of *HR*-carbon. So the ELNES of *HR*-carbon together with those of amorphous carbon, diamond, and graphite can explain the experimental ELNES very well. The comparison of theoretical and experimental Raman spectra is shown in Fig. 6. Previous experiments confirmed that the experimental sample is a mixture of n-diamond and other carbon allotropes. So it is difficult to distinguish the Raman bands of n-diamond from the experimental Raman spectrum. Because the diamond-like carbon allotropes have characteristic peaks in the range of ~1300–1400 cm^−1^ which are located in the range of Raman bands of diamond^54^. However, the *HR*-carbon has three independent characteristic peaks at ~1300 cm^−1^ which can match the experimentally observed three peaks at about 1350 cm^−1^. These also confirm that *HR*-carbon is the most possible candidate structure of n-diamond.

While the graphite transforms to *sp*^3^-hybridized carbon allotropes such as diamond and cold-compressed graphite phase, the hardness of materials enhances much more. So it is worth expecting that *HR*-carbon has high hardness among compressed graphite phases. The calculated bulk modulus (*B*_0_) of *HR*-carbonis 427 GPa which is very close to that of diamond (466 GPa). The Vickers hardness *H*_v_ of *HR*-carbon (80 GPa), estimated by the Chen’s hardness model^55^, is much larger than that of c-BN (~62 GPa). However, it is smaller than that of diamond (~95 GPa). *HR*-carbon has very similar atomic arrangement to that of diamond except the *C*_3_ isosceles triangle rings. So the hardness gap of *HR*-carbon and diamond could be attributed to the unique *C*_3_ isosceles triangle rings. To confirm the effect of *C*_3_ isosceles triangle rings for structural stability, we remove the C_3_ isosceles triangle rings from the crystal structure of *HR*-carbon. This new structure was named as modulated *HR*-carbon (see supplementary Figure S1 and Table S1). We check the mechanical and dynamical properties of modulated *HR*-carbon. The bulk modulus of modulated *HR-carbon* is only 344 GPa which is much smaller than that of *HR*-carbon. So due to unique *C*_3_ isosceles triangle rings in the *C* layer of *HR*-carbon, the *HR*-carbon has stronger incompressibility. We also calculated the phonon dispersion of modulated *HR-carbon* to compare with that of *HR*-carbon Fig. 2(b). The red dashed line of the phonon spectrum is the imaginary phonon mode of modulated *HR*-carbon. The imaginary frequencies at *H* and *Γ* points indicate that the *C*_3_ isosceles triangle rings are very important for the dynamic stability of *HR*-carbon (see Supplementary Figure S1).

In an effort to assess the effect of *C*_3_ isosceles triangle rings for the hardness of *HR*-carbon, we calculated the Mulliken overlap population (MOP) and bond length which can give a quantitative description for the covalent bonds of *HR*-carbon as summarized in Table I. The average bond length and MOP of *HR*-carbon is 1.53 Å and 0.81, respectively, which is comparable with those of diamond (1.531 Å, 0.75). It is noteworthy that the bond length of *C*_3_ isosceles triangle rings is 1.53 Å which is equal to the average bond length. However, the MOP of *C*_3_ isosceles triangle rings is only 0.61 which is much smaller than the average MOP of *HR*-carbon. So the bond strength of *C*_3_ isosceles triangle rings is weaker than that of the other bonds in *HR*-carbon. The bond strength of *C*_3_ isosceles triangle rings is not the mainly reasons for the high hardness of *HR*-carbon. However, it can be found that the *bond*3 has the smallest bond length (1.508 Å) and largest MOP (0.88), indicating the bond strength of *bond*3 is the strongest one in *HR*-carbon. It makes the *C*_3_ isosceles triangle ring stable between Layer *A* and *B* and limits the mobility of Layer *A* and *B* along *a* and *b* directions, resulting in high hardness of *HR*-carbon.

## Conclusion

In conclusion, we have found a novel diamond-like carbon allotrope, *HR*-carbon, which is a likely candidate structure for n-diamond. The simulated XRD, Raman and ELNES of *HR-*carbon can reproduce the experimental results. *HR*-carbon is a semiconductor with an incompressible atomic arrangement. It is stable over graphite above 14.2 GPa. This allotrope possesses high hardness (80 GPa) and high bulk modulus (427 GPa), which are comparable to those of diamond. Unique *C*_3_ isosceles triangle rings are very important for the stability and hardness of *HR*-carbon.

## Methods

Using the USPEX method, the variable-cell structure prediction of n-diamond was performed at 10, 20, 30, 50 and 100 GPa, respectively^50^,^51^,^52^. The simulation cell containing 2 ~ 20 carbon atoms were selected. The structural relaxation were performed within the density functional theory, carried out within the Vienna *ab initio* simulation package (VASP)^56^,^57^. The projector augmented wave method was used^58^. The 2*s*^2^2*p*^2^ electrons are treated as valence electrons. The local density approximation (LDA) was employed^59^. The tested plane-wave cutoff energy was taken as 1100 eV. A Gamma-point-centered *k*-mesh of 7 × 7 × 2 *k*-point sampling was used for the calculations. The geometries were optimized when the remanent Hellmann-Feynman forces on the ions are less than 0.01 eV/Å. The ELNES, Raman and Mulliken population calculations were performed by CASTEP code^60^. The ELNES calculation was performed within the supercell-core-excited approach. A supercell containing 64 atoms was used to avoid the interaction of adjacent core holes. The phonon spectra were calculated using the direct supercell method, performed by PHONON software.

## Additional Information

**How to cite this article**: Li, D. *et al.* *Ab initio* structure determination of n-diamond. *Sci. Rep.* **5**, 13447; doi: 10.1038/srep13447 (2015).

## Supplementary Material

Supplementary Information

## Figures and Tables

**Table 1:** The bond length (*d*) and Mulliken overlap population (*MOP*) of bonds in *HR*-carbon.

***Bond***	***MOP***	***d*** **(Å)**
*Bond*1 (layer A or B)	0.83	1.533
*Bond*2 (between layer A and B)	0.79	1.557
*Bond*3 (between layer A/B and C)	0.88	1.508
*Bond*4 (layer C)	0.61	1.530
