# Supplementary material for: Ab initio structure determination of n-diamond
Source: Sci Rep. 2015 Aug 24;5:13447. doi: 10.1038/srep13447 (PMC4547140; doi:10.1038/srep13447)
Supplement: Supplementary Information [file srep13447-s1.pdf]

Supplementary information

Ab initio structure determination of n-diamond

Da Li[1,2], Fubo Tian[1], Binhua Chu[1], Defang Duan[1], Xiaojing Sha[1], Yunzhou Lv[1], Huadi Zhang[1], Nan Lu[2], Bingbing Liu[1] & Tian Cui[1*]

[1]State Key Lab of Superhard Materials, College of Physics, Jilin University, Changchun 130012, P. R. China,

[2]State Key Lab of Supramolecular Structure and Materials, College of Chemistry, Jilin University, Changchun 130012, P. R. China.

Correspondence and requests for materials should be addressed to T.C. (cuitian@jlu.edu.cn)

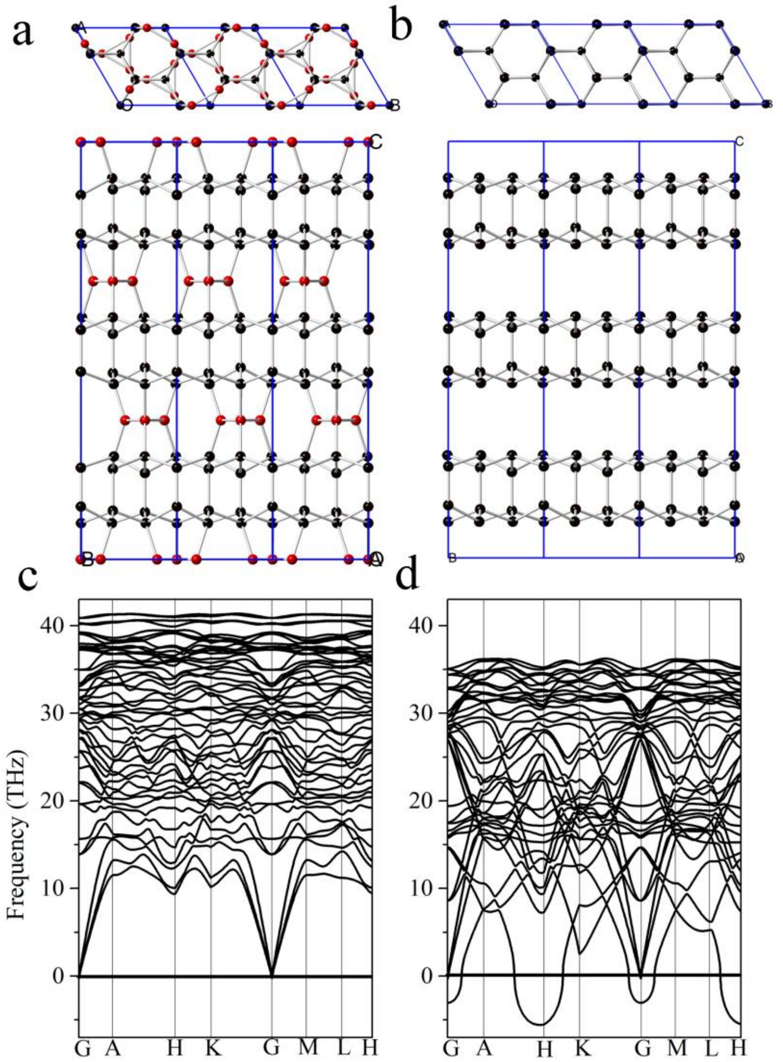

Figure S1 (a) the crystal structures of *HR*-carbon and (b) modulated *HR*-carbon and (c) the phonon dispersion curves of *HR*-carbon and (d) modulated *HR*-carbon.

Table S1 The space groups, lattice parameters, and wyckoff positions of *HR*-carbon and modulated *HR*-carbon.

Structure	Space group	Parameters	Wyckoff Positions	(x, y, z)
HR-carbon	*R*32	$a = b = 4.31$ Å<br>$c = 16.29$ Å<br>$\alpha = \beta = 90$ °<br>$\gamma = 120$ °	18*f*	(0.667, 0.009, 0.247)
				(0.871, 0.538, 0.333)
			6*c*	(0.667, 0.333, 0.114)
				(0.000, 0.000, 0.127)
				(0.333, 0.667, 0.117)
Modulated HR-carbon	*R32*	$a = b = 4.31$ Å<br>$c = 16.29$ Å<br>$\alpha = \beta = 90°$<br>$\gamma = 120$ °	18*f*	(0.667, 0.009, 0.247)
			6*c*	(0.667, 0.333, 0.114)
				(0.000, 0.000, 0.127)
				(0.333, 0.667, 0.117)
